# Carbon Cage Nanosensors for Selective Detection of Toxic Gas Molecules

**DOI:** 10.1021/acsomega.6c00676

**Published:** 2026-03-13

**Authors:** Laith A. Algharagholy, Qusiy H. Al-Galiby, Víctor M. García-Suárez, Isam Nghaimesh Taeb

**Affiliations:** † Department of Physics, College of Science, 717165 University of Sumer, Al-Rifaee, Thi-Qar 64005, Iraq; ‡ Department of Physics, College of Education, University of Al-Qadisiyah, Al Diwaniyah 58002, Iraq; § Departamento de Física, Universidad de Oviedo & CINN, Oviedo 33007, Spain

## Abstract

The detection of toxic substances is of utmost importance in diverse fields, from industry to medicine, and their precise discrimination could lead to significant advances in all of them. Here, we propose a novel nanosensor with a carbon cage structure that can effectively discriminate between 5 types of toxic gas molecules, namely AsH_3_, SO_3_, H_2_S, H_2_Se, and H_2_Te. We use first-principles simulations to design such a device and study its electronic and transport properties in the absence or presence of the molecules. The density of states and transmission of the combined systems change dramatically for all cases, which translates into different currents and Seebeck coefficients that clearly allow to effectively discriminate all of them. These results pave the way for the design of more precise and sensitive nanoscale devices that can lead to improved detection and discrimination of various substances.

## Graphical abstract

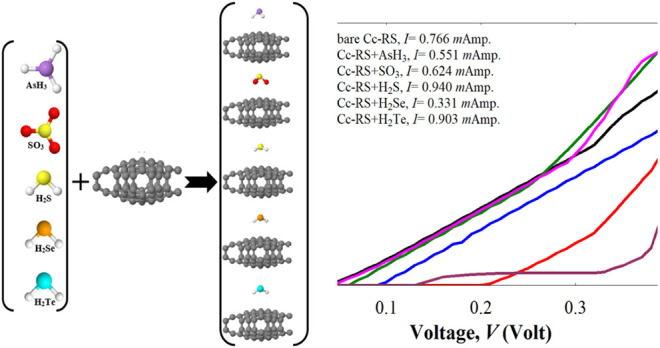

## 1 Introduction

The urgent need for rapid and accurate detection of odorless, hazardous, and toxic gases in exposed environments has driven scientists and research groups to develop new nanomaterials, device concepts, and strategies for designing, managing, and optimizing precise sensor chips. The annual release of large amounts of pollutants into the air, water, and soil has serious consequences for the health of humans, animals, and plants worldwide. Consequently, the accurate identification, control, and elimination of hazardous gas pollutants have become major global challenges.^1–3^

Hazardous gases may occur in numerous environments, including industrial sites (chemical, petroleum, coal, gas, mining), storage facilities, confined spaces, vehicles, sewage systems, waste disposal areas, residential spaces, and even war zones.^4^ The rapid expansion of modern lifestyles, combined with economic, technological, and industrial development that demands significant energy resources, exposes people to a wide variety of air pollutants. Moreover, industrial waste and byproducts resulting from accelerated industrialization are highly hazardous to the environment and human health, thereby requiring continuous monitoring and control.^5–7^

Air pollution is a leading cause of a broad range of health problems, many of which demand costly emergency treatment, and several pollutants can be fatal upon inhalation.^4,8–11^ According to the World Health Organization (WHO), air pollution contributes to asthma exacerbations and increased respiratory infections, particularly in children. It has also been linked to higher morbidity and mortality from cardiovascular diseases, stroke, chronic respiratory disorders, and cancer.^12,13^ WHO reported that in 2016, outdoor air pollution caused approximately 4.2 million deaths, while indoor (household) air pollution accounted for an additional 3.6 million deaths.^12^ Therefore, the detection and identification of toxic gases and hazardous substances provide substantial benefits not only to industry but also to public health and daily life.^10^ To prevent or mitigate poisoning incidents and related accidents, it is crucial to have systems capable of rapidly and accurately detecting, identifying, alerting, and monitoring hazardous gases.

Various techniques have been developed for precise hazardous gas detection, including spectrophotometry, chromatography, mass spectrometry, and Fourier-transform infrared spectroscopy.^12,14,15^ However, these methods are often time-consuming, costly, and unsuitable for real-time analysis.^12^ In contrast, electrochemical and electrical detection methods—such as potentiometric, amperometric, and conductometric techniques^7^—have shown great promise and significant recent progress.^16–19^ Furthermore, device miniaturization enables nanoscale electrical transduction,^16^ leading to reduced costs and lower power consumption compared to conventional approaches. Label-free detection of small molecules, including hazardous gases, is also advantageous as it removes the need for chemical modification or analyte separation.^20–25^ Low-dimensional materials such as nanotubes,^21,22,26–30^ graphene,^8,31–34^ and fullerenes^35–38^ have been extensively explored for selective, single-molecule gas sensing.

In this work, we present a novel electrochemical nanosensor design resembling a rhombus-shaped carbon cage, referred to hereafter as *Cc*-RS (Figure 1). This structure possesses high quantum confinement and strong surface/interface effects, which can significantly improve selective detection performance (in contrast to 2D materials, such as graphene, that do not benefit as much from quantum confinement, which could favor specific detection mechanisms).^39^ We explore the ability of *Cc*-RS to selectively detect several hazardous gases, focusing on five common toxic analytes: arsine (AsH_3_),^40^ sulfur trioxide (SO_3_),^41,42^ hydrogen sulfide (H_2_S),^10^ hydrogen selenide (H_2_Se), and hydrogen telluride (H_2_Te).^43^ These gases pose severe risks to human health and the natural environment, making their detection and selective sensing essential.

**Figure 1:**
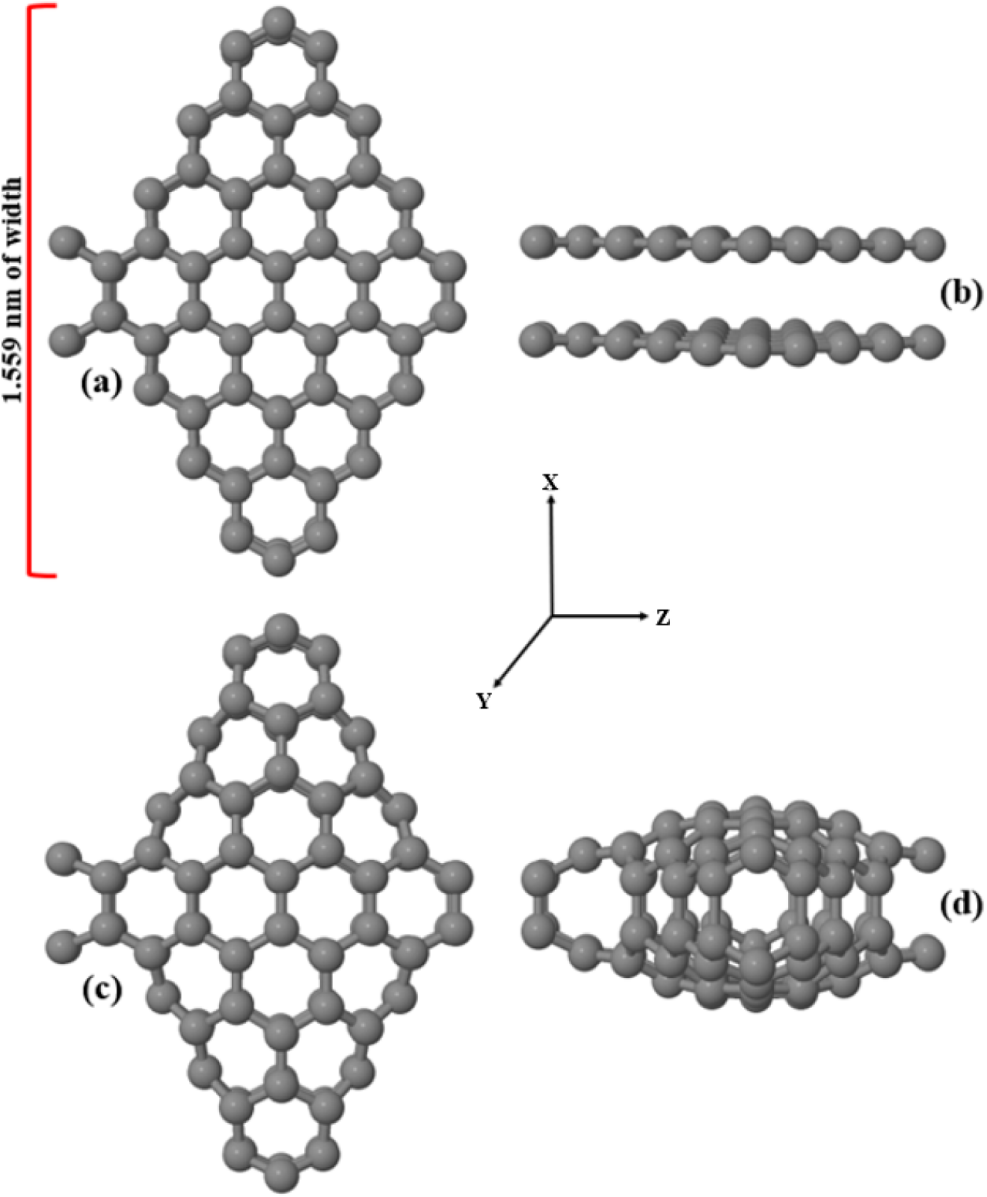
(a and b) Front and side views of one unrelaxed cell of *Cc*-RS, respectively. (c and d) Front and side views of one relaxed cell of *Cc*-RS, respectively. The cells are periodic along the Z direction and finite along the X and Y directions.

## 2 Computational Tools

The fabrication of the nanosensor begins with the sculpturene method,^44^ through which we obtain from the unrelaxed configuration that can be seen in Figure 1a–b the final relaxed *Cc*-RS structure shown in Figure 1c–d. The coordinates of such relaxed structure are also provided in the SI (Table S1). The fundamental principle of the sculpturene approach is to cut or sculpt specific shapes from bilayer graphene or heterostructures and allow them to reconstruct globally into unique molecular forms. This process can also be carried out experimentally^21,22^ by cutting single- or few-layer graphene using techniques such as scanning tunneling microscope (STM) lithography,^45^ chemical etching,^46^ or sonochemical methods.^47^

The sculpturene methodology enables the creation of novel nanoscale architectures composed of pure sp^2^-bonded carbon and allows the design of unprecedented heterostructures. Examples include the spontaneous reconstruction of carbon nanotubes (CNTs) from bilayer graphene nanoribbons (biGNRs) or heterobilayer nanoribbons (hbiNRs), forming sp^2^-bonded T-shaped or cross-branched CNTs. Another type of sculpturene arises from carved bilayer graphene that reconstructs into new sp^2^-bonded carbon cages, which are topologically distinct from fullerenes and remain stable against atomic-scale defects.^44^ These structures form when the width of the bilayer graphene nanoribbon is approximately 3 nm or less. Conversely, when the width exceeds 3 nm, the structures remain flat, although edge reconstruction still occurs to maximize sp^2^ bonding.^44^ In addition, this technique allows precise control of CNT chirality by positioning them at specific locations within a device and connecting them ohmically to electrodes in a planar configuration.^48^

To construct the rhombus-like nanosensor, we began by sculpting a rhombus shape from an AA-stacked zigzag bilayer graphene nanoribbon (ZbiGNR) with a width of 1.559 nm, as shown in Figure 1a–b. The initial distance between layers was 3.2 Å, which properly leads to the formation of the final structure (notice that if that distance was too large, the final structure may not be formed). We then performed density functional theory (DFT) calculations using the SIESTA code^49^ and the generalized gradient approximation (GGA-PBE) as the exchange–correlation functional.^50^ It is worth noting that several previous studies have reported that GGA-PBE and local density approximation (LDA-CA) functionals tend to underestimate the energy gap (*E*_g_) when compared to experimental results.^51–58^ To ensure accurate characterization of the electronic properties, all final calculations were therefore carried out using the hybrid GGA-HSE06 functional, which has proven highly reliable for predicting the electronic behavior of a wide range of materials^59^ and has also been used in the calculation of other sensing systems.^60^

Core electrons were treated with norm-conserving pseudopotentials, and the valence states were described using a double-ζ polarized basis set. The real-space grid was generated using a plane-wave cutoff energy of 300 Ry. To avoid interactions between neighboring structures, we applied a vacuum spacing of 60 Å along the X and Y directions, while periodic boundary conditions were imposed along the Z direction. Furthermore, to prevent interactions between toxic gas molecules in adjacent periodic images, we ensured a separation distance along the Z axis ranging from 10.11 Å to 10.32 Å in all relaxed combined systems. Since the system has no overlaps along the X and Y directions, all calculations are performed with an integration of the Brillouin zone sampled with a 1 × 1 × 30 k-grid Monkhorst–Pack mesh. Both the bare *Cc*-RS structure and the corresponding complexes (*Cc*-RS+AsH_3_, *Cc*-RS+SO_3_, *Cc*-RS+H_2_S, *Cc*-RS+H_2_Se, and *Cc*-RS+H_2_Te) were fully relaxed until the forces on all atoms were smaller than 0.01 eV/Å.

To more clearly assess the stability of the structure we also calculated the cohesive energy, the phonon spectra and carried out ab initio molecular dynamics (AIMD) simulations at 300 and 400 K on both bare *Cc*-RS and the combined systems. The AIMD simulations were performed utilizing LAMMPS, using the AIREBO potential for C–C interactions and Lennard-Jones potentials for weak interactions between C atoms and adsorbate molecules (AsH_3_, SO_3_, H_2_S, H_2_Se, and H_2_Te). All systems were evolved for a total duration of 2000 fs using the Velocity Verlet integration algorithm with a time step of 0.5 fs, ensuring accurate propagation of atomic positions and velocities. Additionally, uniaxial tensile strains of 1% along the *z*-axis were examined to investigate mechanical response of the systems.

The electronic transmission coefficients, *T*(*E*), were calculated using the GOLLUM quantum transport code,^61^ based on the electronic structure obtained from SIESTA. GOLLUM calculates the transmission from the equilibrium electronic structure and uses different approximations^61^ to obtain the transmission at finite bias, from which the current (*I*) is calculated by using the Landauer–Büttiker formalism for quantum transport:^62^

1 $$I=\frac{e}{h}\int T\left(E\right)\left[f\left(E-\mu_{L}\right)-f\left(E-\mu_{R}\right)\right]dE$$

where *e* = |*e*| is the elementary charge, *h* is Planck’s constant, *T*(*E*) is the electronic transmission probability (calculated using the GOLLUM code), *f*(*E* – μ) is the Fermi–Dirac distribution, and μ_L_ and μ_R_ are the electrochemical potentials of the left and right leads, respectively. From the transmission it is also possible to calculate the Seebeck coefficient (*S*) as follows:^63^

2 $$S=\frac{-1}{eT}\frac{L_{1}}{L_{0}}$$

Where $L_{n}=\int_{-\infty }^{+\infty }{\left(E-E_{F}\right)}^{n}T\left(E\right)\left(\frac{-\partial f}{\partial E}\right)dE$ .

To identify the most stable configurations of the optimized structures, we computed the binding energy (BE) for each molecule interacting with the *Cc*-RS surface. The BE values were calculated using eq 1), which accounts for the basis-set superposition error (BSSE):^64,65^

3 $$\mathrm{BE}=E^{\left(\mathrm{Cc}‐\mathrm{RS}+\mathrm{gas}\right)}-\left(E^{\left(\mathrm{Cc}‐\mathrm{RS}\right)}+E^{\left(\mathrm{gas}\right)}\right)$$

where *E* ^(*Cc*‑RS+gas)^ represents the total energy of the combined systems (*Cc*-RS+AsH_3_, *Cc*-RS+SO_3_, *Cc*-RS+H_2_S, *Cc*-RS+H_2_Se, and *Cc*-RS+H_2_Te), while *E* ^(*Cc‑*RS)^ and *E* ^(gas)^ correspond to the total energies of the isolated subsystems.

## 3 Results and Discussion

### 3.1 Electronic Properties of Bare *Cc*-RS

The main objective behind designing the unique *Cc*-RS geometry is to demonstrate its suitability for the detection and selective sensing of various toxic gas molecules, namely AsH_3_, SO_3_, H_2_S, H_2_Se, and H_2_Te. The structure of the bare device is illustrated in Figures 1 and 2. Notably, it contains a single type of defect—an eight-membered (octagonal) ring—while the remainder of the framework consists of hexagons. When toxic gas molecules are adsorbed onto this surface, the interaction induces local variations in the potential energy of the *Cc*-RS, which subsequently alter the charge distribution within the structure. These modifications lead to significant changes in its electronic and transport properties, as will be discussed below.

**Figure 2:**
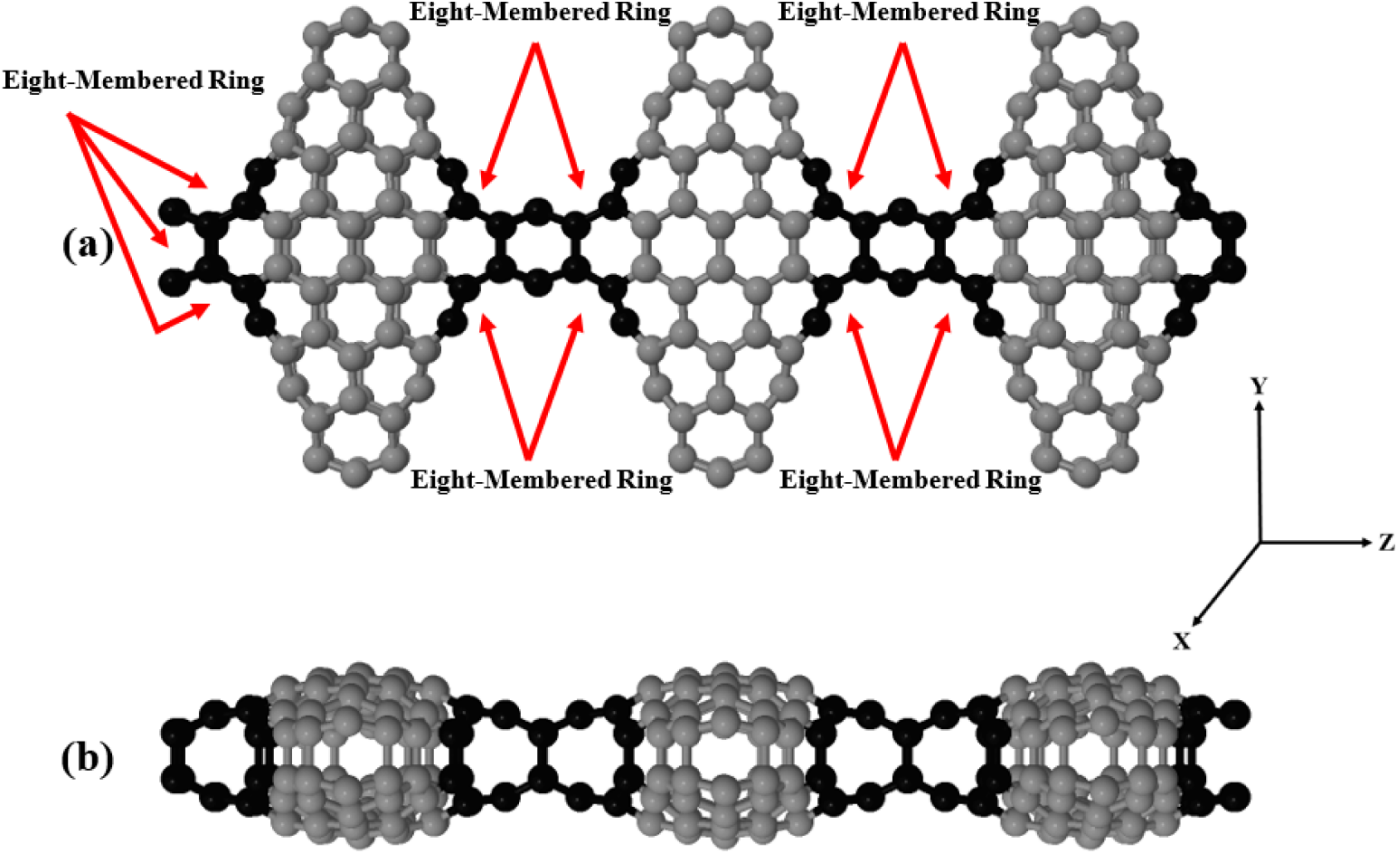
(a and b) Front and side view of the *Cc*-RS supercell, which contains four eight-membered (octagon) rings.

The stability of the folded (relaxed) *Cc*-RS structure shown in Figure 1d, relative to the unfolded (unrelaxed) configuration shown in Figure 1b, can be evaluated by comparing their total energies. The total energy of the folded structure is lower (−15,442.176 eV) than that of the unfolded structure (−15,341.147 eV), which is expected since the unfolded configuration contains dangling bonds that increase its overall energy. This confirms that the folded geometry is energetically more favorable. The phonon spectra of bare *Cc*-RS show no negative frequencies, except some very small in a tiny interval around the G point (see Figure S3), probably due to numerical instabilities, which confirms the dynamic stability of the system. Also, the time evolution of the bare *Cc*-RS at a temperature of 400 K is shown in Figure S1a; as can be seen, the variation of the cohesive energy is very small during the simulation period, which confirms the thermal stability of the structure.

To gain a deeper understanding of the electronic behavior of the pristine *Cc*-RS, we first analyze its density of states (DOS), shown in Figure 3. The DOS represents the number of available electronic states per unit energy near the Fermi energy (*E*_F_); integrating the DOS up to *E*_F_ yields the total number of electrons. This quantity is fundamental in describing the electronic characteristics of molecules and materials.^31^

**Figure 3:**
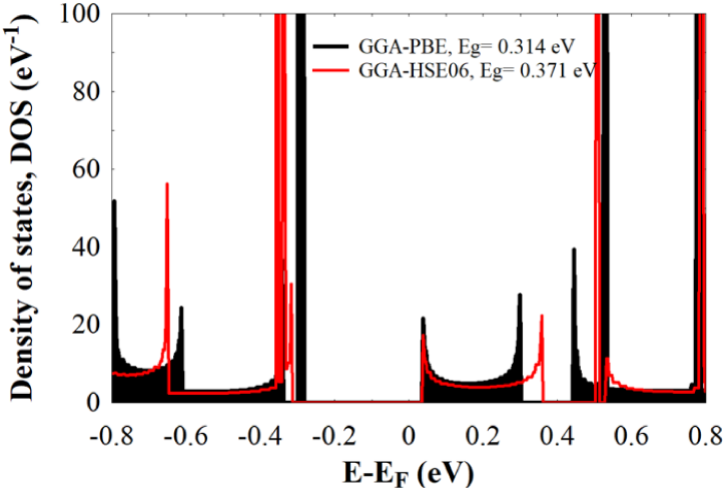
DOS of bare *Cc*-RS calculated with a GGA-PBE functional (black), and with a GGA-HSE06 functional (red).

For comparison, two exchange–correlation (XC) functionals—GGA-PBE and GGA-HSE06—were employed to calculate the electronic properties. As shown in Figure 3, the *Cc*-RS behaves as a semiconductor, with an energy gap (*E*_g_) of 0.314 eV for GGA-PBE and 0.371 eV for GGA-HSE06. The narrower gap obtained with the GGA-PBE functional aligns well with previous studies,^66–71^ which reported that GGA-PBE tends to underestimate the bandgap of semiconductors and insulators. Additionally, other features such as bandwidth exhibit slight variations depending on the chosen functional, highlighting the importance of an accurate selection of the exchange–correlation functional to reliably describe the electronic properties of the system.

### 3.2 Electronic and Transport Properties of *Cc*-RS with Toxic Gas Molecules

After obtaining the relaxed geometry of the *Cc*-RS system, the toxic gas molecules were positioned on top of the *Cc*-RS surface, as illustrated in Figure 4. This figure shows both the relaxed configurations of the gas molecules and the combined systems (*Cc*-RS+AsH_3_, *Cc*-RS+SO_3_, *Cc*-RS+H_2_S, *Cc*-RS+H_2_Se, and *Cc*-RS+H_2_Te). Apart from these configurations, we considered also other possible adsorption sites and found that the ones used here (the molecule on top of the central part of the structure) were the most stables. Notice as well that the adsorption of the (nonmagnetic) molecules does not give rise to any spin-polarized electronic structure. These systems are also evolved over time using AIMD as shown in Figures S1 and S2. The variation of the cohesive energy is very small in all cases, for both the relaxed structure and also with a uniaxial tensile strain of 1%, like for the bare structure, which confirms the thermal stability of such structures. The phonon spectra show as well no negative frequencies, confirming also the dynamic stability. It should also be noted that, although this exact structure has not been experimentally fabricated, it is well-known that similar carbon cages^72^ can be created and that these can be rebuilt by self-repair of the edges of the bilayer graphene, forming closed edges with sp^2^ bonds.^73–76^

**Figure 4:**
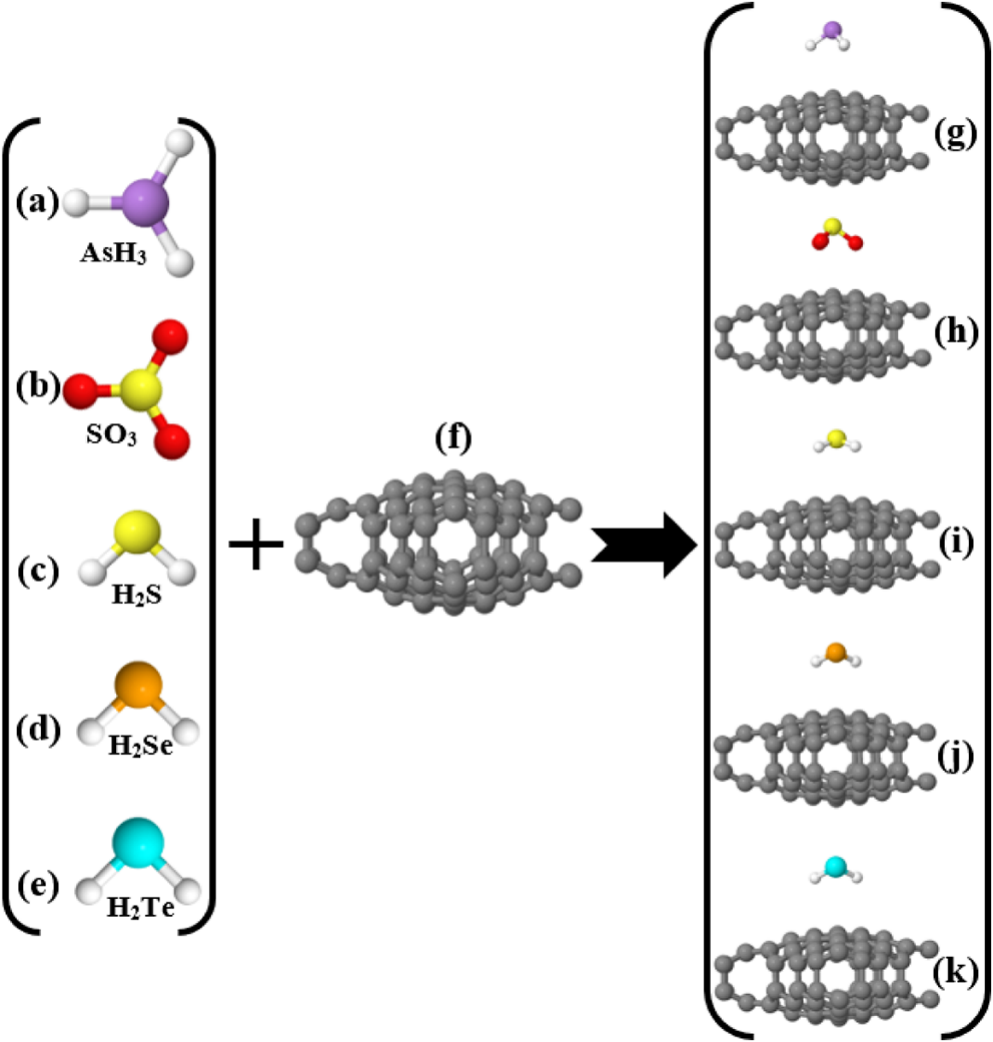
(a–e) The targeted toxic gas molecules AsH_3_, SO_3_, H_2_S, H_2_Se, and H_2_Te respectively, (f) the bare *Cc*-RS unit, and (g–k) the final relaxed structures of the toxic gas molecules on top of *Cc*-RS, i.e., *Cc*-RS+AsH_3_, *Cc*-RS+SO_3_, *Cc*-RS+H_2_S, *Cc*-RS+H_2_Se, and *Cc*-RS+H_2_Te.

The BE results, calculated using eq 2, are summarized in Table 1, and show that all binding energies are negative, indicating that the formation of these systems is exothermic and can occur spontaneously without additional energy input. Among them, the lowest binding energies were found for H_2_S (−0.376 eV), H_2_Se (−0.398 eV), and H_2_Te (−0.382 eV), while AsH_3_ (−0.543 eV) and SO_3_ (−0.491 eV) exhibited the highest values. This trend is expected since AsH_3_ and SO_3_ are larger molecules that interact more strongly with the surface. These energies are moderate: they are large enough to ensure stable adsorption and measurable changes in the electronic and transport properties, yet sufficiently small to permit desorption through thermal activation or exposure to vacuum. Importantly, these binding energies fall below the threshold typically associated with chemisorption (>1 eV), implying that the interaction is predominantly physisorptive or weakly chemisorptive. This means that the carbon cage maintains its structural integrity upon adsorption, without forming irreversible bonds that would compromise long-term operation (as can also be seen in Figure 4). Since none of the adsorption events involve structural deformation, bond cleavage in the cage, or large barrier-induced trapping, the sensing device is expected to return to its initial state after desorption. Molecules such as H_2_S, H_2_Se, and H_2_Te, which show the lowest binding energies (≈−0.38 eV), should detach most easily, enabling a rapid reset of the sensor. Even the strongest binders (AsH_3_ and SO_3_) can remain within the desorption range accessible by standard regeneration procedures in nanosensor operation such as modest heating (<150–200 °C) or purging.

**Table 1:** Energy Gap (*E*_g_), Binding Energy (BE), Charge Transfer (CT, Given in Electrons per Molecule; Positive Values of CT Mean That *Cc*-RS Gives Charge to the Molecules), Current Change at 0.4 V and Largest Values (Positive or Negative) of the Seebeck Coefficient for AsH_3_, SO_3_, H_2_S, H_2_Se, and H_2_Te Molecules on Top of *Cc*-RS

Structure →	bare *Cc*-RS	*Cc*-RS+AsH_3_	*Cc*-RS+SO_3_	*Cc*-RS+H_2_S	RS+H_2_Se	*Cc*-RS+H_2_Te
*E*_g_ (eV)	0.371	0.495	0.476	0.262	0.454	0.503
BE (eV)	-	–0.543	–0.491	–0.376	–0.398	–0.382
CT	-	+0.515 *e*	–0.006 *e*	+0.363 *e*	+0.523 *e*	–0.421 *e*
*I* (mA) at 0.4 V	0.766	0.551	0.624	0.940	0.331	0.904
*S* (μV/K) at *E*_F_	–209.890	733.672	385.512	282.645	–465.759	–252.297

To gain deeper insight into the electronic behavior of the combined systems, we evaluated the charge transfer (CT) between the molecules and the *Cc*-RS using Mulliken charge population analysis (Table 1). The results show that the CT values fluctuate between positive and negative signs. For *Cc*-RS+AsH_3_, *Cc*-RS+H_2_S, and *Cc*-RS+H_2_Se, the CT values are positive, indicating electron transfer from the *Cc*-RS to the adsorbed molecules. Conversely, for *Cc*-RS+SO_3_ and *Cc*-RS+H_2_Te, the CT is negative, implying electron transfer from the gas molecules to the *Cc*-RS. Therefore, AsH_3_, H_2_S, and H_2_Se act as electron donors, while SO_3_ and H_2_Te behave as electron acceptors. Since the binding energies are consistent with physisorption, the charge transfer is mainly due to electrostatic polarization between the molecules and the carbon cage, rather than orbital hybridization or strong chemical bonding.

To further assess the sensing capability of *Cc*-RS toward these gases, we analyzed the density of states (DOS) of the combined systems, shown in Figure 5. The plots reveal that molecular adsorption substantially modifies the electronic structure of the *Cc*-RS, as reflected by the variations in the energy gap (*E*_g_), summarized as well in Table 1. In particular, adsorption of H_2_S reduces the band gap from 0.371 eV (bare *Cc*-RS) to 0.262 eV, while adsorption of AsH_3_ (*E*_g_ = 0.495 eV), SO_3_ (*E*_g_ = 0.476 eV), H_2_Se (*E*_g_ = 0.454 eV), and H_2_Te (*E*_g_ = 0.503 eV) increases it. These variations in *E*_g_ provide a preliminary indication that the *Cc*-RS structure can discriminate between different toxic gas molecules based on their distinct electronic signatures.

**Figure 5:**
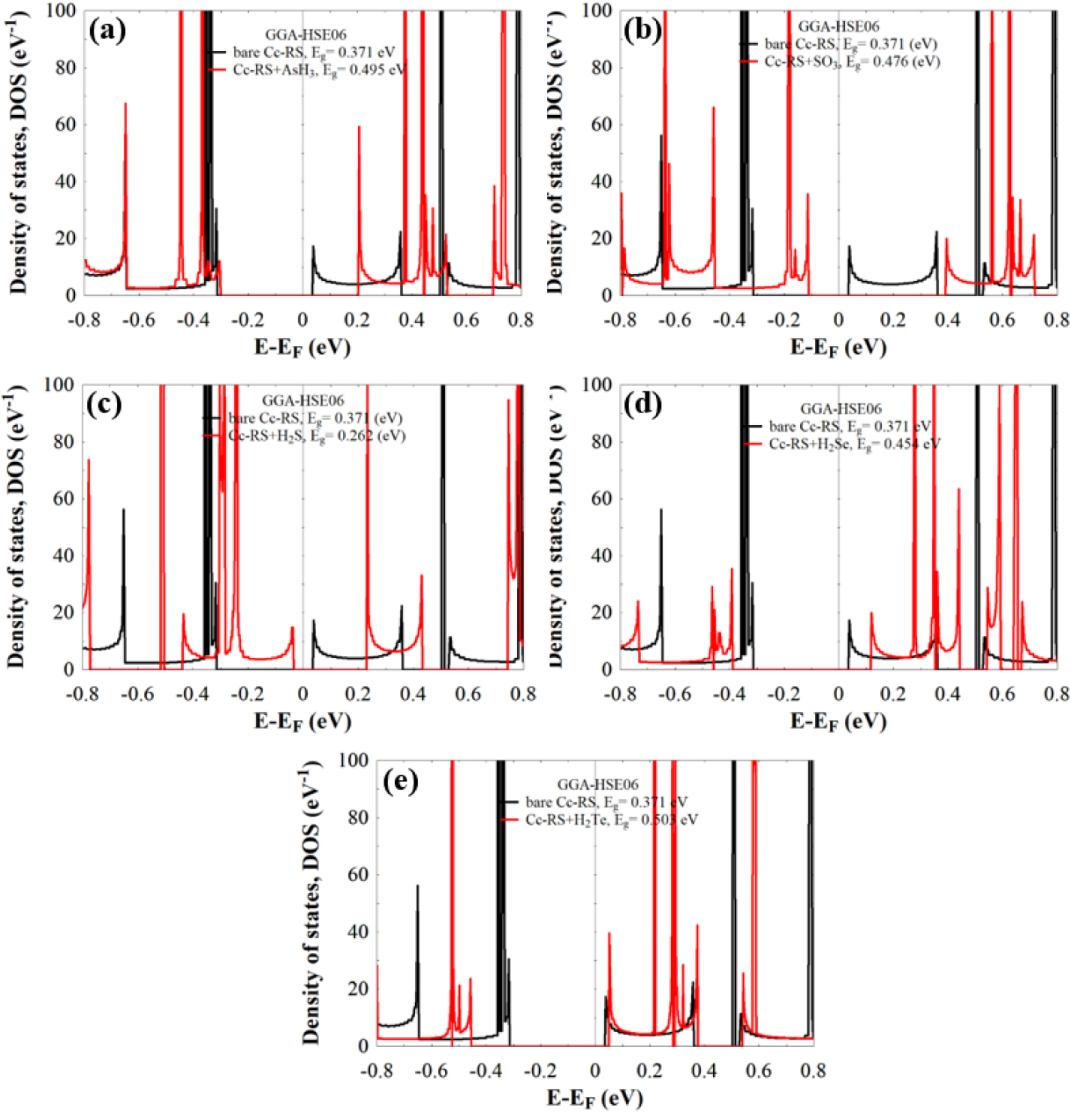
(a–e) The DOS of *Cc*-RS+AsH_3_, *Cc*-RS+SO_3_, *Cc*-RS+H_2_S, *Cc*-RS+H_2_Se, and *Cc*-RS+H_2_Te, respectively. In all subfigures the black line represents the DOS of the bare *Cc*-RS system. For all calculations, a mesh of 1 × 1 × 30 k-points was used. *E*_F_ is the predicted Fermi energy value given by DFT.

Additional confirmation of this selective sensing capability was obtained by computing the electronic transmission coefficients, *T*(*E*), for the bare and combined systems. Figure 6 depicts the device setup, where left and right leads are connected to the scattering region (extended molecule). Notice that the leads need to have at least two units (cells) to define the intracell and intercell Hamiltonians that GOLLUM needs to compute the transport properties, while the scattering region needs in this case (periodic system) only one unit cell because there are no surfaces or “molecules” (i.e., structures that are different from the electrodes). Figure 7 presents the corresponding transmission spectra. Each combined system exhibits a distinct transmission profile, consistent with the DOS results, but with added information regarding the number of open channels (bands) and the influence of molecular adsorption on them.

**Figure 6:**
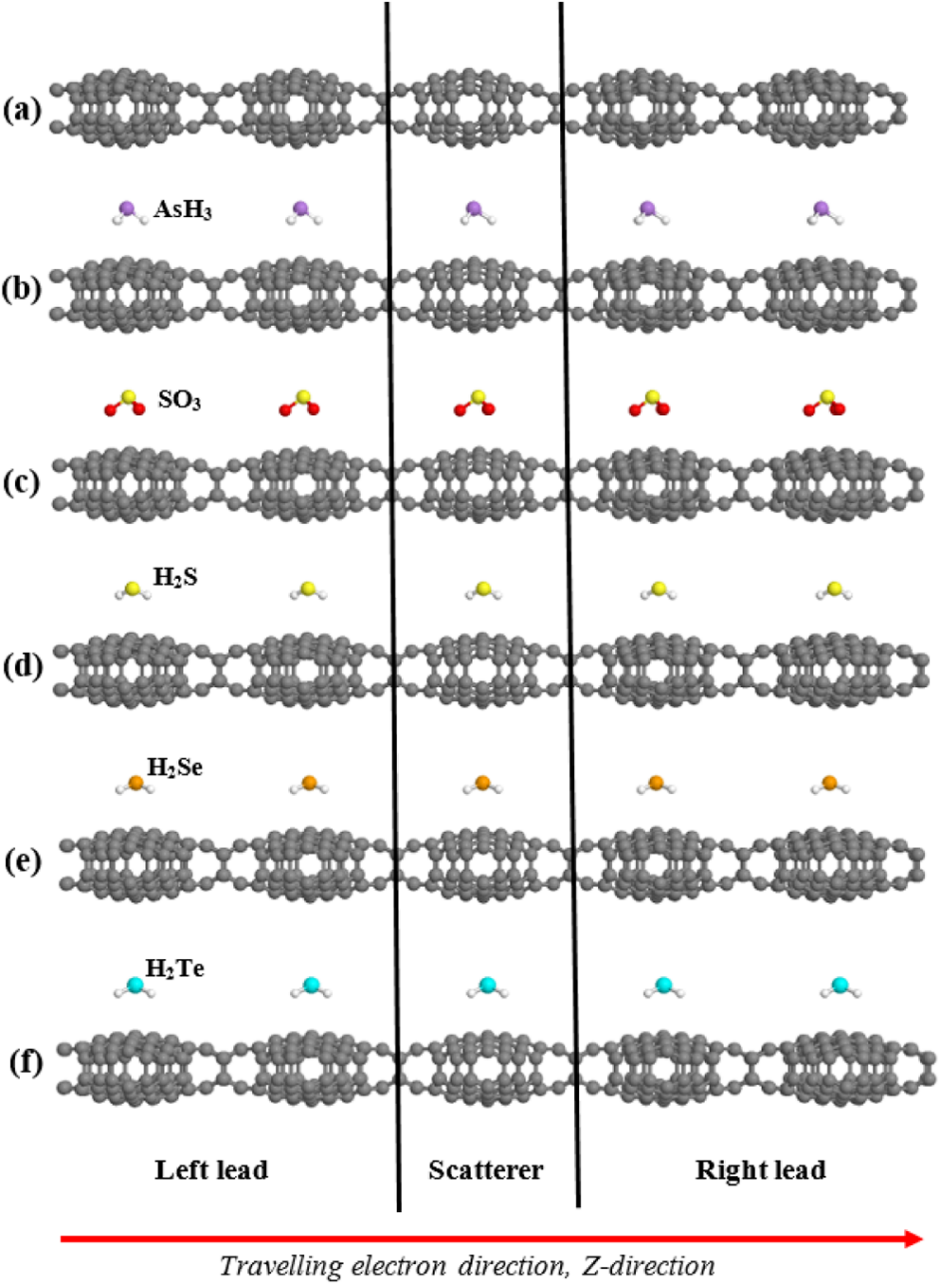
(a–f) *Cc*-RS devices with AsH_3_, SO_3_, H_2_S, H_2_Se, and H_2_Te molecules on top of them.

**Figure 7:**
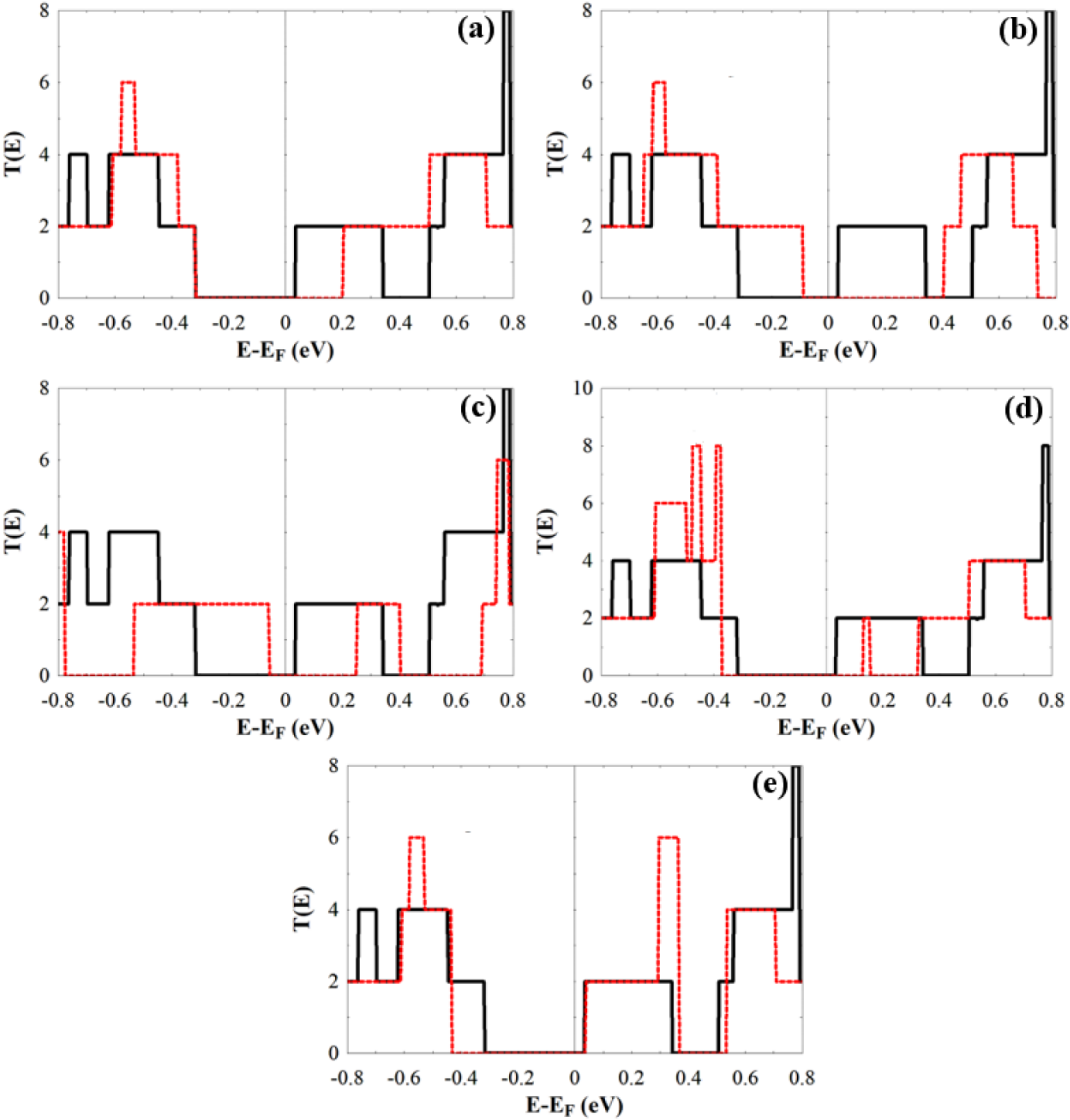
(a–e) The *T*(*E*) of the *Cc*-RS+AsH_3_, *Cc*-RS+SO_3_, *Cc*-RS+H_2_S, *Cc*-RS+H_2_Se, and *Cc*-RS+H_2_Te, respectively. The black lines in all subfigures is for the bare *Cc*-RS.

To evaluate the sensing performance under operating conditions, we also calculated and compared the room-temperature current (*I*) of the *Cc*-RS in the absence and presence of the different gas molecules (Figure 8). The current was obtained using eq 1.

**Figure 8:**
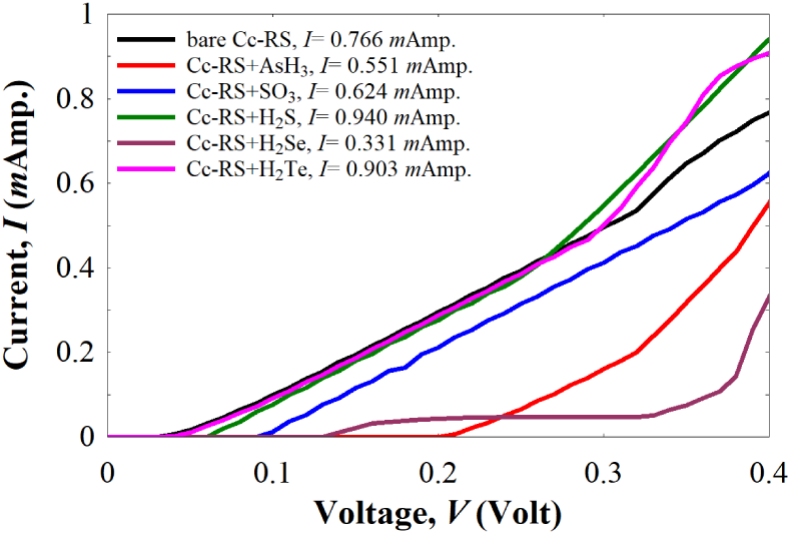
Room temperature current *I* of the bare *Cc*-RS and *Cc*-RS+AsH_3_, *Cc*-RS+SO_3_, *Cc*-RS+H_2_S, *Cc*-RS+H_2_Se, and *Cc*-RS+H_2_Te.

As shown in Figure 8, the current of the bare *Cc*-RS at 0.4 V is 0.766 mA, while those for *Cc*-RS+AsH_3_, *Cc*-RS+SO_3_, *Cc*-RS+H_2_S, *Cc*-RS+H_2_Se, and *Cc*-RS+H_2_Te are 0.551 mA, 0.624 mA, 0.940 mA, 0.331 mA, and 0.904 mA, respectively. These results are comparable to other nanoscale systems used to sense different types of molecules.^21,23^ The variations of the current values, given also in Table 1 at a voltage of 0.4 V, demonstrate that each adsorbed molecule significantly alters the current response, and despite some similarities (e.g., between H_2_S and H_2_Te), the differences are sufficient to allow discrimination between them. Previous studies^77–81^ have also shown that extremely small currents (in the nanoampere or picoampere range) can be reliably measured, confirming that these current changes are experimentally detectable even at lower voltages. This highlights the potential of the *Cc*-RS system for effective and selective detection of toxic gases.

Furthermore, our results show that the transmission spectra *T*(*E*) in all cases display an asymmetric shape around the Fermi level (*E*_F_), which can enhance the Seebeck coefficient (*S*) of the combined systems compared to the bare *Cc*-RS, since this quantity depends on the presence of such asymmetric features inside the thermal window given by the derivative of the Fermi distribution function. We computed then *S* at room temperature (300 K). The calculated *S* values, shown in Figure 9 and given also in Table 1, confirm this trend. At *E*_F_, *S* is −209.890 μV/K for the bare *Cc*-RS, while for the combined systems *Cc*-RS+AsH_3_, *Cc*-RS+SO_3_, *Cc*-RS+H_2_S, *Cc*-RS+H_2_Se, and *Cc*-RS+H_2_Te, the corresponding values are 733.672 μV/K, 385.512 μV/K, 282.645 μV/K, −465.759 μV/K, and −252.297 μV/K, respectively. These values change moderately with temperature in an operating temperature range between 200 and 400 K due to thermal broadening, as shown in figure S4 (Seebeck coefficient calculated at 400 K), while the trends remain essentially the same, indicating the robustness of the Seebeck coefficient in these systems.

**Figure 9:**
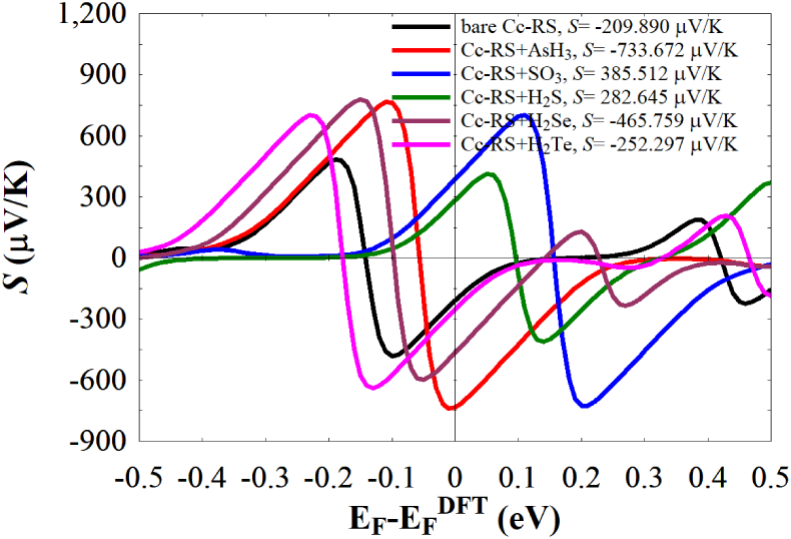
Seebeck coefficient *S* calculated at room temperature of bare *Cc*-RS and *Cc*-RS+AsH_3_, *Cc*-RS+SO_3_, *Cc*-RS+H_2_S, *Cc*-RS+H_2_Se, and *Cc*-RS+H_2_Te shown in Figure 6, evaluated at $E_{F}=E_{F}^{\mathrm{DFT}}$ , where $E_{F}^{\mathrm{DFT}}$ represents the value of the Fermi energy given by DFT.

These large values can be found in other nanostructures due to the sharpness of some features that enter the thermal window^82^ and are similar as well to those found in other types of periodic nanostructures.^83^ These findings demonstrate that molecular adsorption can markedly enhance the thermoelectric response of the system. Moreover, the sign of *S* also changes depending on the adsorbed molecule: bare *Cc*-RS, *Cc*-RS+AsH_3_, *Cc*-RS+H_2_Se, and *Cc*-RS+H_2_Te exhibit p-type behavior (negative *S*), while *Cc*-RS+SO_3_ and *Cc*-RS+H_2_S display n-type characteristics (positive *S*). Notice that the sign depends on the features that enter the thermal window as the Fermi energy shifts. If there appears a feature at high energies that increases the transmission or at low energies that decreases it, then it gives a negative contribution to the Seebeck coefficient. If, however, there is a feature at high energies that decreases the transmission or at low energies that increases it, then it gives a positive contribution to the Seebeck coefficient. Notice as well that when the Seebeck coefficient is positive, holes are the majority carriers diffusing toward the cold side, and the material therefore exhibits p-type behavior. Conversely, a negative Seebeck coefficient implies that electrons dominate the transport toward the cold side, corresponding to n-type behavior. Regarding the maxima (positive *S*) and minima (negative *S*) observed in the evolution of the thermopower in Figure 9, a maximum of the thermopower for a given Fermi energy is reached when the product of the negative derivative of the Fermi function multiplied by the transmission below the Fermi level is maximized, and/or when this product is minimized above the Fermi level. The opposite occurs for a minimum: it is reached when the product of the negative derivative of the Fermi function multiplied by the transmission above the Fermi level is maximized, and/or when this product is minimized below the Fermi level. Conversely, *S* is zero or very small when the transmission below and above the Fermi level within the thermal window is the same or very similar.

Overall, our results reveal that the electronic and transport properties —including DOS, *T*(*E*), current (*I*), and the magnitude and sign of *S*— are strongly influenced by the specific analyte adsorbed on the *Cc*-RS surface. The combined variations in these parameters provide distinctive electronic fingerprints for each gas, allowing precise and reliable discrimination. This confirms that the proposed *Cc*-RS nanosensor represents a powerful and efficient platform for the selective detection of toxic gas molecules.

## 4 Conclusions

We have demonstrated that carbon cage nanostructures fabricated via the sculpturene method constitute a promising platform for selective toxic gas sensing. After structural relaxation, the cages remain stable and exhibit two accessible adsorption surfaces. Their periodic arrangement displays a small band gap, confirming semiconducting behavior suitable for sensing applications.

The gas adsorption is exothermic and induces charge transfer between the analyte and the carbon cage, with molecules acting as either donors or acceptors. The calculated binding energies indicate sufficiently strong interactions to enable selective detection, while remaining low enough to ensure reversible adsorption without structural degradation. This balance supports the potential reusability of the device.

Adsorption-driven modifications in the density of states alter the band gap and propagate to the transmission spectra, generating molecule-specific signatures in both the electrical current and Seebeck coefficient. These distinct electronic responses enable clear discrimination among different gases.

Notice as well that, while the results show that toxic gas molecules can be distinguished and sensed, the same nanocage structures should also work for common background gases such as N_2_, O_2_, H_2_O, CO_2_, CH_4_, N_2_O, and others. In particular, since the largest changes are produced by polar molecules such as those in the present study, it should be possible to clearly discriminate and sense polar molecules like H_2_O and N_2_O. Nonpolar molecules such as N_2_, O_2_, CO_2_, and CH_4_ would be more challenging, since the absence of a dipole does not provide an electrostatic field that can affect the electronic states of the cage. However, the likely adsorption of such compounds on the carbon surface, similar to previous studies,^84^ should also affect the electronic states of the cage and lead to sizable changes in the electronic states and therefore in the current and Seebeck coefficient.

Future work should focus on experimental realization, building upon existing advances in the fabrication of graphene-derived nanostructures and carbon nanoarchitectures. Recent progress in bottom–up synthesis of graphene nanoribbons, defect-engineered graphene, and three-dimensional carbon frameworks suggests that controlled sculpting and reconstruction of carbon lattices is experimentally feasible at the nanoscale.^85–88^ Techniques such as electron-beam patterning, chemical vapor deposition (CVD), and on-surface synthesis have demonstrated precise structural control in related systems,^89,90^ providing a realistic pathway toward the fabrication of sculpturene-based cages. In addition, developments in contacting low-dimensional carbon nanostructures with metallic electrodes and integrating them into microelectronic platforms indicate that device-level implementation is achievable. Systematic evaluation under realistic operating conditions—including temperature, humidity, and competing background gases—will be essential to bridge the gap between theoretical predictions and deployable sensing technologies.

## Supplementary Material



## Notes

Additional calculations (cohesive energies, phonon dispersion relations, and Seebeck coefficient at 400 K); data (*Cc*-RS coordinates in xyz format) (PDF)
