# Supplementary material for: Carbon Cage Nanosensors for Selective Detection of Toxic Gas Molecules
Source: ACS Omega. 2026 Mar 13;11(11):18427–37. doi: 10.1021/acsomega.6c00676 (PMC13019188; doi:10.1021/acsomega.6c00676)
Supplement: Supplementary file 1 [file ao6c00676_si_001.pdf]

Supporting information

Carbon cage nanosensors for selective detection of toxic gas molecules

Laith A. Algharagholy[1*], Qusiy H. Al-Galiby[2], Víctor M. García-Suárez [3*], Isam Nghaimesh Taeb[1]

[1]Department of Physics, College of Science, University of Sumer, Al-Rifaee, 64005, Thi-Qar, Iraq.
[2]Department of Physics, College of education, University of Al-Qadisiyah, Al Diwaniyah 58002, Iraq.
[3]Departamento de Física, Universidad de Oviedo & CINN, Oviedo, 33007, Spain.
[*]Corresponding author: l.algharagholy@gmail.com, garciavictor@uniovi.es

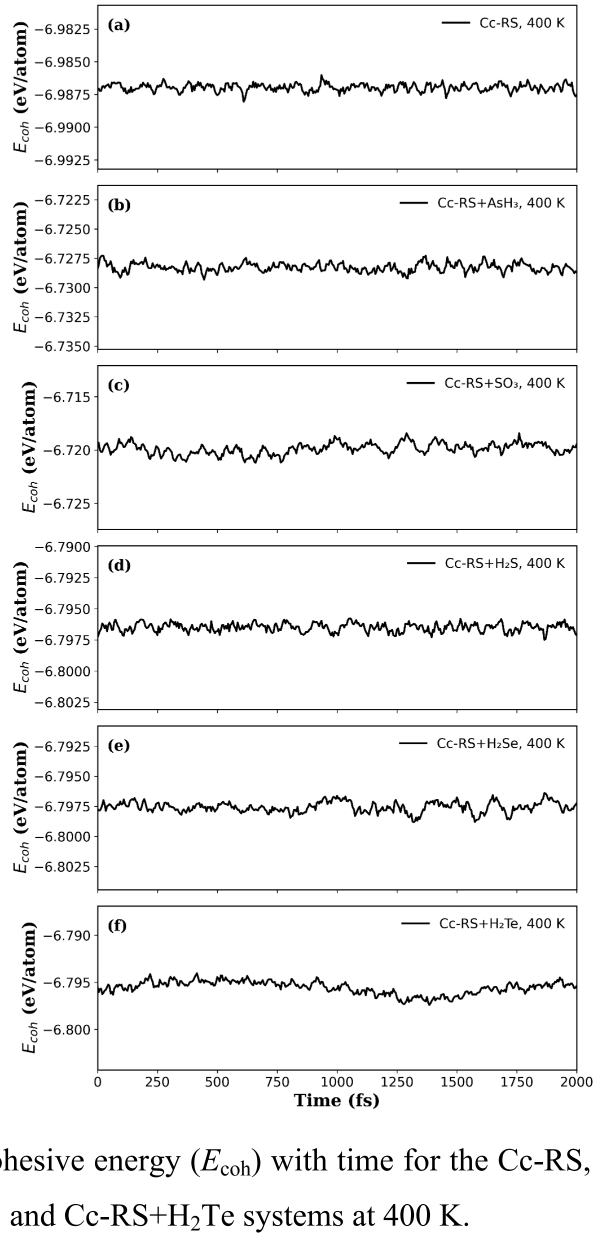

Figure S1: Variation of cohesive energy (E_{coh}) with time for the Cc-RS, Cc-RS+AsH_3, Cc-RS+SO_3, Cc-RS+H_2S, Cc-RS+H_2Se, and Cc-RS+H_2Te systems at 400 K.

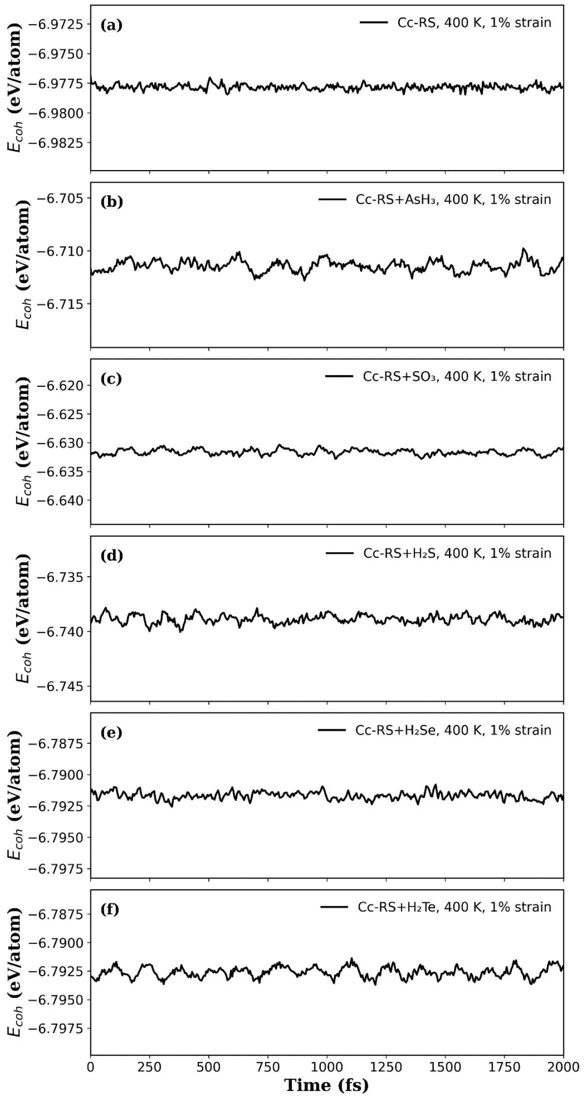

Figure S2: Variation of cohesive energy (E_{coh}) with time for the Cc-RS, Cc-RS+AsH_3, Cc-RS+SO_3, Cc-RS+H_2S, Cc-RS+H_2Se, and Cc-RS+H_2Te systems under 1% uniaxial tensile strain along the z-axis at 400 K.

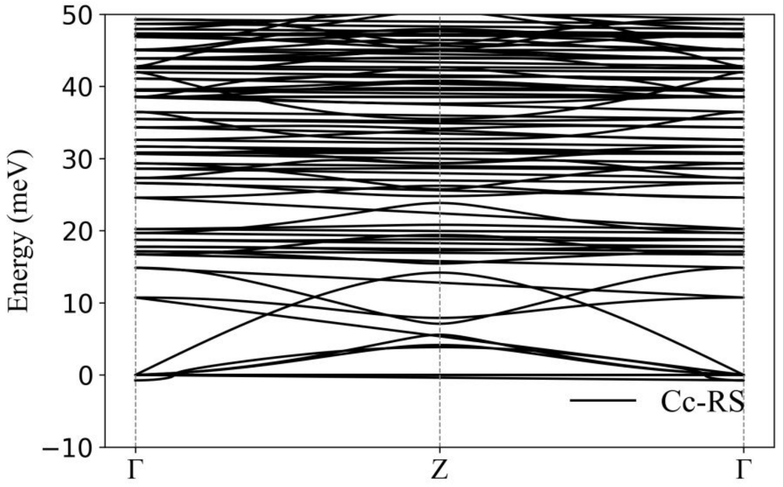

Figure S3: Phonon dispersion relations for the bare structure Cc-RS.

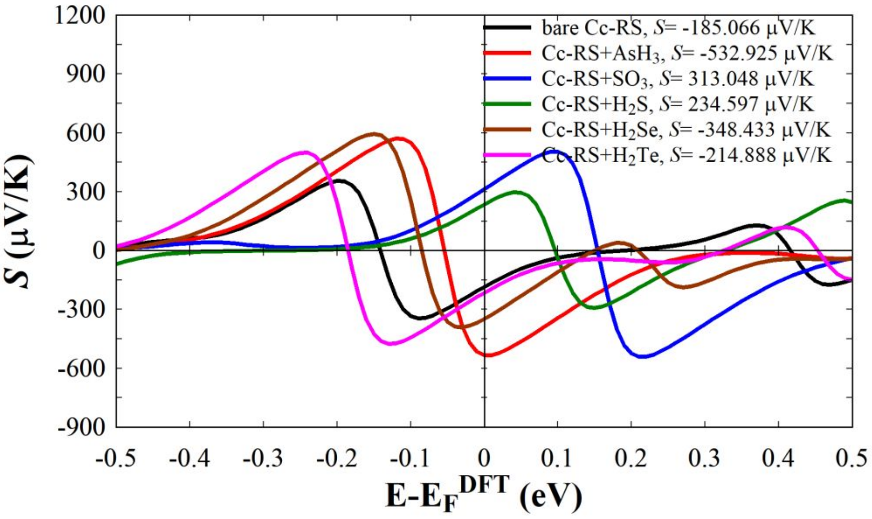

Figure S4: The Seebeck coefficient S calculated at 400 K of bare Cc-RS and Cc-RS+AsH_3, Cc-RS+SO_3, Cc-RS+H_2S, Cc-RS+H_2Se, and Cc-RS+H_2Te shown in Figure 6, evaluated at $E_F = E_F^{DFT}$, where E_F^{DFT} represents the value of the Fermi energy given by DFT.

Table S1: Coordinates of bare Cc-RS (in xyz format).

100

C 11.768160001 10.484590001 5.502143001

C 11.463780001 10.001740001 6.783823001

C 13.316250002 10.483320001 5.502038001

C	13.619810002	10.000000001	6.783739001
C	11.768090001	10.484700001	8.065099001
C	13.316260002	10.483470001	8.064984001
C	11.814820001	12.864470002	4.654780001
C	13.273610002	12.863360002	4.654803001
C	11.214420001	11.865290001	5.498856001
C	10.716600001	12.473040001	6.783467001
C	13.872280002	11.863100001	5.498853001
C	14.371060002	12.470070001	6.783431001
C	11.214400001	11.865510001	8.068193001
C	11.814870001	12.864800002	8.912034001
C	13.872290002	11.863320001	8.068098001
C	13.273690002	12.863640002	8.912024001
C	11.818290001	15.069510002	3.541634000
C	11.209690001	14.141370002	4.457057001
C	13.274160002	15.068420002	3.541643000
C	13.880980002	14.139180002	4.457220001
C	10.418920001	14.699180002	5.546709001
C	10.352260001	13.915930002	6.783295001
C	14.672630002	14.695760002	5.546837001
C	14.737950002	13.912320002	6.783337001
C	10.419010001	14.699390002	8.019703001
C	11.209770001	14.141750002	9.109444001
C	14.672770002	14.695900002	8.019763001
C	13.881110002	14.139440002	9.109422001
C	11.818420001	15.069910002	10.024640001
C	13.274200002	15.068850002	10.024750001
C	11.805020001	16.412150002	0.632793900
C	11.210380001	16.869080002	1.898718000
C	13.290290002	16.410930002	0.632869900

C	13.885450002	16.867020002	1.898710000
C	11.055660001	16.211090002	3.144508000
C	10.411480001	16.880040002	4.288041001
C	14.038800002	16.208580002	3.144751000
C	14.683820002	16.876650002	4.288224001
C	10.156240001	16.138850002	5.522971001
C	10.000000001	16.872410002	6.783031001
C	14.937840002	16.134970002	5.523138001
C	15.095450002	16.868360002	6.783143001
C	10.156350001	16.139060002	8.043178001
C	10.411670001	16.880420002	9.278014001
C	14.937960002	16.135160002	8.043252001
C	14.683900002	16.877060002	9.277998001
C	11.055710001	16.211630002	10.421530001
C	11.210460001	16.869490002	11.667350001
C	14.038830002	16.209070002	10.421540001
C	13.885440002	16.867400002	11.667370001
C	11.806660001	18.798440002	0.632887700
C	11.211370001	18.341930002	1.898594000
C	13.291950002	18.797230002	0.632958400
C	13.886460002	18.339720002	1.898592000
C	11.057670001	19.000110002	3.144370000
C	10.412520001	18.332220002	4.287929001
C	14.040890002	18.997800002	3.144647000
C	14.684940002	18.328840002	4.288118001
C	10.158530001	19.073980002	5.522742001
C	10.001160001	18.340920002	6.782937001
C	14.940180002	19.070290002	5.522897001
C	15.096580002	18.336910002	6.783044001
C	10.158710001	19.074200002	8.042986001

C	10.412750001	18.332650002	9.277952001
C	14.940190002	19.070530002	8.043057001
C	14.684960002	18.329230002	9.277921001
C	11.057740001	19.000550002	10.421510001
C	11.211430001	18.342380002	11.667310001
C	14.040890002	18.998290002	10.421460001
C	13.886470002	18.340200002	11.667320001
C	11.822200001	20.140320002	3.541238000
C	11.215170001	21.069450003	4.456443001
C	13.278090002	20.139110002	3.541326000
C	13.886530002	21.067400003	4.456586001
C	10.423640001	20.513150002	5.546207001
C	10.358380001	21.296560003	6.782679001
C	14.677290002	20.509890002	5.546318001
C	14.743880002	21.293370003	6.782731001
C	10.423780001	20.513380002	8.019263001
C	11.215290001	21.069950003	9.109008001
C	14.677350002	20.510180002	8.019336001
C	13.886610002	21.067990003	9.109001001
C	11.822320001	20.140990002	10.024490001
C	13.278130002	20.139740002	10.024520001
C	11.822470001	22.345370003	4.653800001
C	13.281200002	22.344270003	4.653843001
C	11.223610001	23.345430003	5.497693001
C	10.725010001	22.738740003	6.782464001
C	13.881500002	23.343590003	5.497693001
C	14.379360002	22.736160003	6.782475001
C	11.223670001	23.345640003	8.067005001
C	11.822480001	22.345830003	8.911298001
C	13.881510002	23.343820003	8.067037001

C	13.281330002	22.344770003	8.911315001
C	11.779440001	24.725170003	5.500368001
C	11.475710001	25.208600003	6.782279001
C	13.327440002	24.724090003	5.500382001
C	13.631770002	25.207180003	6.782259001
C	11.779380001	24.725580003	8.063350001
C	13.327550002	24.724520003	8.063349001
